# Constructing High‐Performance Inverted Perovskite Solar Cells Using Chiral Organic Molecules

**DOI:** 10.1002/advs.202417550

**Published:** 2025-04-01

**Authors:** Zixuan Shang, Jinbao Han, Hongliang Dong, Mengxi Lv, Qianru Zhang, Zhiqiang Chen, Mingxing Wu, Jinjin Zhao

**Affiliations:** ^1^ Hebei Technology Innovation Center for Energy Conversion Materials and Devices Hebei Key Laboratory of Inorganic Nanomaterials Engineering Research Center of Thin Film Solar Cell Materials and Devices College of Chemistry and Materials Science Hebei Normal University Shijiazhuang Hebei 050024 P.R. China; ^2^ Center for High Pressure Science and Technology Advanced Research Pudong Shanghai 201203 P.R. China; ^3^ Shanghai Key Laboratory of Material Frontiers Research in Extreme Environments (MFree) Shanghai Advanced Research in Physical Sciences (SHARPS) Pudong Shanghai 201203 P.R. China

**Keywords:** chiral organic molecules, passivation molecules, perovskite solar cells, S‐MBACl

## Abstract

Chiral molecules have shown potential in passivating perovskite solar‐cell interfaces and boosting charge transport and have drawn significant research interest. However, the specific passivation mechanisms of different chiral structures on perovskite films and their photoelectric effects require further investigation. In this study, chiral *R*‐, *S*‐, and *rac*‐methylbenzylammonium chloride (MBACl) molecules are used to address interface defects. *S*‐MBACl exhibits the strongest chelation and passivation effects. Kelvin probe force microscopy results show that *S*‐MBACl increases the surface potential differences between dark and illuminated states by 227%, from 39.67 to 129.91 mV, and enhances electron–hole separation. Consequently, the power conversion efficiency (PCE) of *S*‐MBACl‐modified devices is 24.07%, which is 109% times that of the pure perovskite sample. The PCE of unencapsulated *S*‐MBACl‐modified perovskite solar cells remains at 89% of the initial value after aging at 25 °C for 2400 h in the N_2_ atmosphere. This study provides valuable insights for future studies on chiral passivation molecules.

## Graphical abstract

This study presents a strategy for passivating defects at perovskite interfaces using chiral molecules. A comparison between the chiral R‐, S‐, and rac‐MBACl molecules reveals that S‐MBACl exhibits the strongest chelating ability and defect passivation effect on the perovskite surface. Finally, PSCs based on S‐MBACl modification achieve a PCE of 24.07%.

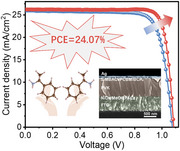

## 1 Introduction

Owing to the exceptional photoelectric properties of metal halide perovskites, perovskite solar cells (PSCs) have achieved a certified power conversion efficiency (PCE) of up to 26% in just over a decade.^[^ ^1^ ^]^ Although significant achievements have been made, considerable room for improvement to achieve the Shockley–Queisser theoretical limit of 33.7% remains.^[^ ^2^ ^]^ This gap is mainly attributed to defects such as ion substitution, energy gaps, and vacancies in perovskite materials; these defects not only hinder the effective extraction of photogenerated charges but also impair the PCE and long‐term stability.^[^ ^3^ ^]^ Therefore, introducing passivation materials to reduce interfacial defects has become a key strategy for enhancing the performance of PSCs.^[^ ^4^ ^]^ Among the various passivation materials, chiral molecules exhibit completely different optical and electrical properties owing to their small structural differences and have attracted considerable attention from researchers.^[^ ^5^ ^]^ For instance, Chen et al. used chiral α‐methylphenylethylamine cations (*S*‐/*R*‐/*rac*‐MBA) to enhance hole transport in FASnI_3_‐based PSCs; they discovered that introducing these cations can lead to the formation of 2D/3D films, which aid in achieving energy‐level alignment and efficient charge transfer at the interface.^[^ ^5^ ^]^ Chen et al. used chiral *S*‐, *R*‐, and rac‐ibuprofen molecules for the post‐treatment of perovskite films and simultaneously repaired the interface defects, and adjusted the interface‐band alignment.^[^ ^6^ ^]^ Duan et al. used two chiral molecules, *R*‐ and *S*‐methylbenzylammonium iodide, at the interface between the perovskite and electron‐transport layers to improve the mechanical stability and defect passivation, thus enhancing PSC stability under thermal‐cycling and damp‐heat conditions.^[^ ^7^ ^]^ Gao et al. post‐treated 3D perovskite surfaces with chiral *β*‐methylphenylethylammonium iodide to reduce defect‐assisted recombination and achieve favorable energy‐level alignment.^[^ ^8^ ^]^ Although the use of chiral molecules in passivating PSC interfaces and promoting charge extraction and transport has recently progressed, the relationship between the structure of chiral molecules and the photoelectric properties of perovskite films requires further investigation.

To further explore the intrinsic relationship between chiral molecules and the photoelectric properties of perovskite films, chiral *R*‐, *S*‐, and *rac*‐methylbenzylammonium chloride (MBACl) molecules are innovatively used in this study to regulate the interface defects between the perovskite and charge‐transport layers. Research has shown that functional groups on chiral molecules can effectively bind uncoordinated Pb^2+^ on the perovskite surface and form strong hydrogen bonds. *S*‐MBACl exhibits the strongest chelating ability on the perovskite surface, providing the best defect‐passivation effect. Characterizations are performed to study the interaction mechanism between *S*‐MBACl and the perovskite surface. *S*‐MBACl significantly reduces the defect density in the perovskite films and increases the carrier lifetime and diffusion length. *S*‐MBACl also promotes the energy‐level alignment and carrier extraction between the perovskite and charge‐transport layers. Finally, the PCE of *S*‐MBACl‐modified devices is 24.07%, which is 109% times that of the pure perovskite sample. Furthermore, the PCE of unencapsulated *S*‐MBACl‐modified PSCs remains at 89% of the initial PCE after aging at 25 °C for 2400 h in the N_2_ atmosphere.

## 2 Results and Discussion

### 2.1 Effect of Chiral Molecules on the Microstructure of Perovskite

**Figure 1:**
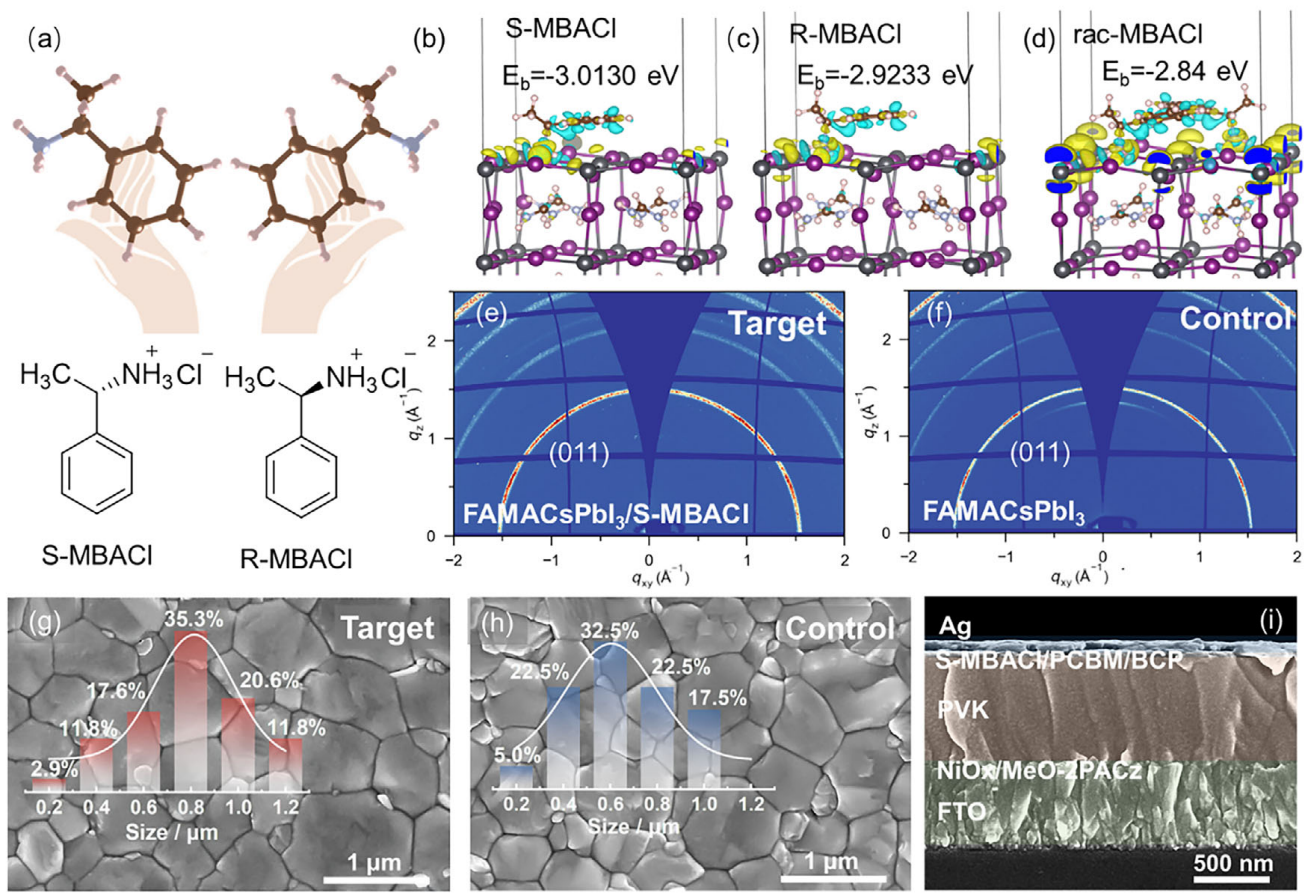
Influence of chiral molecules on the microstructure of perovskite. a) Molecular structures of S‐MBACl and R‐MBACl. Binding energies of b) S‐MBACl, c) R‐MBACl, and d) rac‐MBACl with the perovskite lattices (at the same molecular coverage density). 2D‐GIWAXS results of perovskite films e) with and f) without S‐MBACl modification. Top‐view SEM images of perovskite films g) with and h) without S‐MBACl modification. i) Cross–sectional SEM image of a perovskite device modified with S‐MBACl.

Chiral molecules significantly affect the micromorphology of perovskites. **Figure** **1**a shows the structures of the chiral *S*‐MBACl and *R*‐MBACl molecules. *Rac*‐MBACl is a racemic mixture of equal amounts of *R*‐MBACl and *S*‐MBACl. The amine group (NH_3_^+^) in the chiral molecules can passivate the uncoordinated Pb^2+^ and A‐site cation vacancy defects on the perovskite surface.^[^ ^9^ ^]^ Additionally, Cl^−^ can regulate the crystallization of perovskite, enhancing structural stability.^[^ ^10^ ^]^ The CD spectrum (Figure S1, Supporting Information) reveals that S‐MBACl and R‐MBACl exhibit distinct mirror‐image circular dichroism signals ≈295 nm, whereas rac‐MBACl shows no significant signal. This confirms the successful synthesis and enantiomeric purity of the chiral materials.^[^ ^11^ ^]^ To quantify the optical activity, specific rotations were measured for S‐MBACl, R‐MBACl, and rac‐MBACl, yielding values of −0.38°, 0.66°, and 0.18°, respectively. To quantify the optical activity, specific rotations were measured for S‐MBACl, R‐MBACl, and rac‐MBACl, yielding values of −0.38°, 0.66°, and 0.18°, respectively. Positive specific rotation values indicate dextrorotatory behavior, while negative values indicate levorotatory behavior.^[^ ^12^ ^]^ The specific rotation of rac‐MBACl lies between those of the enantiomers, consistent with its nature as a racemic mixture.

To clarify the interaction between the chiral organic molecules and perovskite, we utilize density functional theory (DFT) to calculate the chemical binding energies (*E*_b_) of *S‐*MBACl, *R‐*MBACl, and *rac‐*MBACl on the perovskite surface. The results show that the binding energy (*E*_b_) of *S‐*MBACl on the perovskite surface is −3.01 eV, which is lower than that of *R*‐MBACl (−2.92 eV). This indicates that *S*‐MBACl binds more stably to the perovskite surface. At the same molecular coverage density, the *E*_b_ of *rac*‐MBACl is −2.84 eV, attributed to hydrogen bonding that distorts the PbI_6_⁴⁻ octahedral framework on the surface and changes the orientation of A‐site cations near the surface.^[^ ^13^ ^]^ According to the differential charge density, the H atom of the ─NH₃ group forms a hydrogen bond with I on the perovskite surface, causing electrons to accumulate between H^…^I. Compared with Figure 1d,c presents a more extensive region of yellow electron accumulation. This observation suggests that hydrogen bonding between S‐MBACl and the perovskite is stronger, which is consistent with the lower E_b_ value obtained. Based on the inverted device structure of FTO/NiOx/MeO‐2PACz/FAMACsPbI_3_/PCBM/BCP/Ag, the influence of the three chiral ligand molecules on device performance is studied. The statistical data show that the short‐circuit current density (*J*_SC_), open‐circuit voltage (*V*_OC_), filling factor (FF), and PCE increase after modification with chiral ligands (Figure S2, Supporting Information). Figure S3 (Supporting Information) presents the PCEs of perovskite solar cells before and after molecular (S, R, and rac‐MBACl chiral molecules) modification.

Grazing incidence wide‐angle X‐ray scattering (GIWAXS) was employed to examine the crystal structure of the perovskite layer and further investigate the optimization effects of *S‐*MBACl molecules. The GIWAXS data presented in Figure 1e,f reveal an enhancement in the crystallinity of the perovskite film after modification with *S‐*MBACl.^[^ ^14^ ^]^ The scanning electron microscopy (SEM) images in Figure 1g–i depict that the perovskite grains of the target sample significantly increase in size and exhibit a more uniform distribution. This indicates that *S*‐MBACl effectively promotes the growth of perovskite grains and inhibits the formation of defects.^[^ ^15^ ^]^ Furthermore, the cross‐sectional view of the target sample (Figure 1i) displays the vertically aligned growth of the perovskite layer on the substrate, with a thickness of ≈700 nm. This structure is beneficial for suppressing the recombination of charge carriers, thereby further enhancing the device's performance.^[^ ^16^ ^]^ The energy‐dispersive X‐ray spectroscopy(EDS)elemental‐mapping image reveals that the elements in the *S‐*MBACl‐modified perovskite are uniformly distributed (Figure S4, Supporting Information).

### 2.2 Photoelectric Conversion of Chiral‐Modified Perovskite

**Figure 2:**
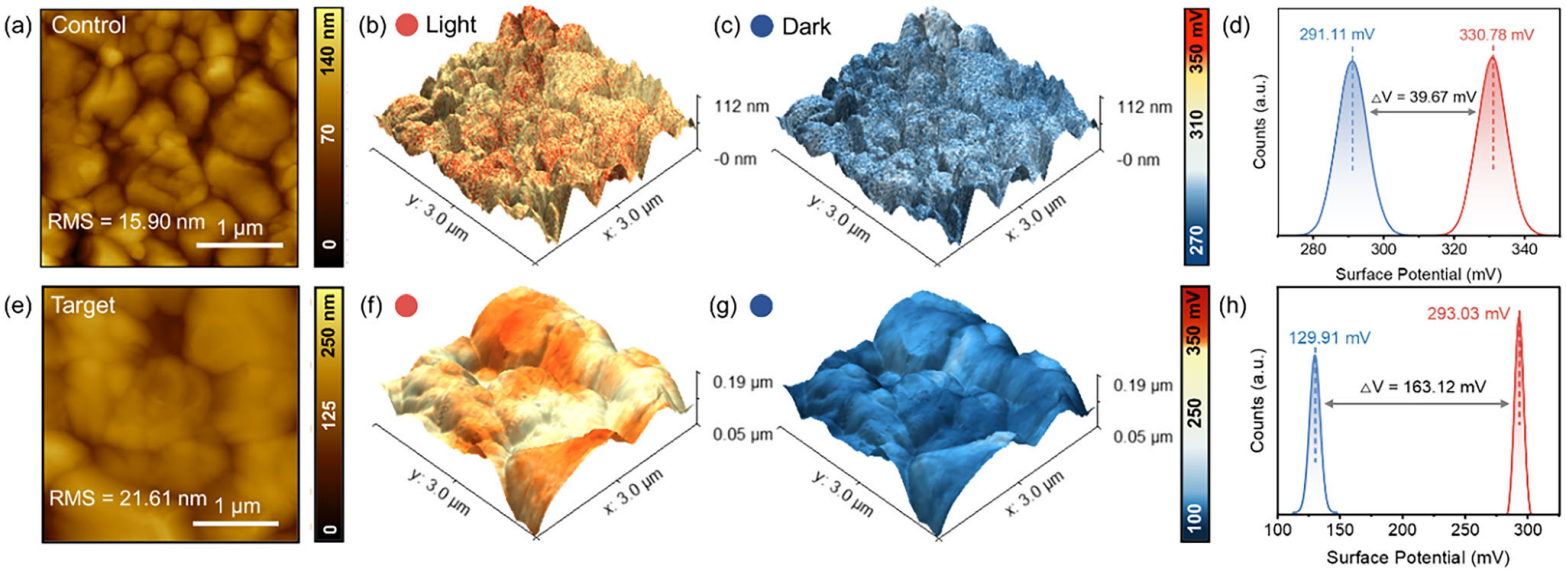
In situ KPFM characterization of perovskite thin films before and after modification under dark and illuminated states. a) Atomic force microscopy (AFM) images of the control perovskite thin film. Overlay of surface potential with morphology under b) illuminated and c) dark states. d) Surface potential change between illuminated and dark states. e) AFM image of the S‐MBACl‐modified perovskite thin film. Overlay of surface potential with morphology under f) illuminated and g) dark states. h) Surface potential change between illuminated and dark states.

The atomic force microscopy (AFM) images in **Figure** **2**a,e quantitatively demonstrate the surface topography changes induced by S‐MBACl. The root mean square (RMS) roughness of the perovskite surface increases from 15.90 to 21.61 nm after S‐MBACl modification. This roughness enhancement is attributed to the enlarged grain size of perovskite grains,^[^ ^17^ ^]^ consistent with the SEM images (Figure 1g,h). To investigate the impact of *S‐*MBACl modification on the photoelectric properties of the perovskite films, Kelvin probe force microscopy (KPFM) was performed to determine the in situ surface potential changes of the control and modified perovskite films under dark and illuminated states. Figure 2b–d clearly shows that the average surface potential of the control sample is 291.11 mV in darkness and increases to 330.78 mV upon light excitation, which is an increase of only 39.67 mV. In contrast, the average surface potential of the *S*‐MBACl‐modified perovskite sample is 129.91 mV in darkness and increases to 293.03 mV upon light excitation, which is an impressive increase of up to 163.12 mV (Figure 2f–h). The variation in the surface potential between the dark and illuminated states of perovskite films directly reflects the generation and transport of photogenerated carriers.^[^ ^18^ ^]^ The introduction of *S*‐MBACl significantly improves the surface potential difference of the perovskite films under light exposure. This enhancement not only facilitates the effective separation of electrons and holes but also strengthens the directional migration ability of charge carriers.^[^ ^19^ ^]^ Therefore, *S*‐MBACl enhances the photoelectric performance of the perovskite films.

### 2.3 Effect of Chiral Materials on the Structure of Perovskite

**Figure 3:**
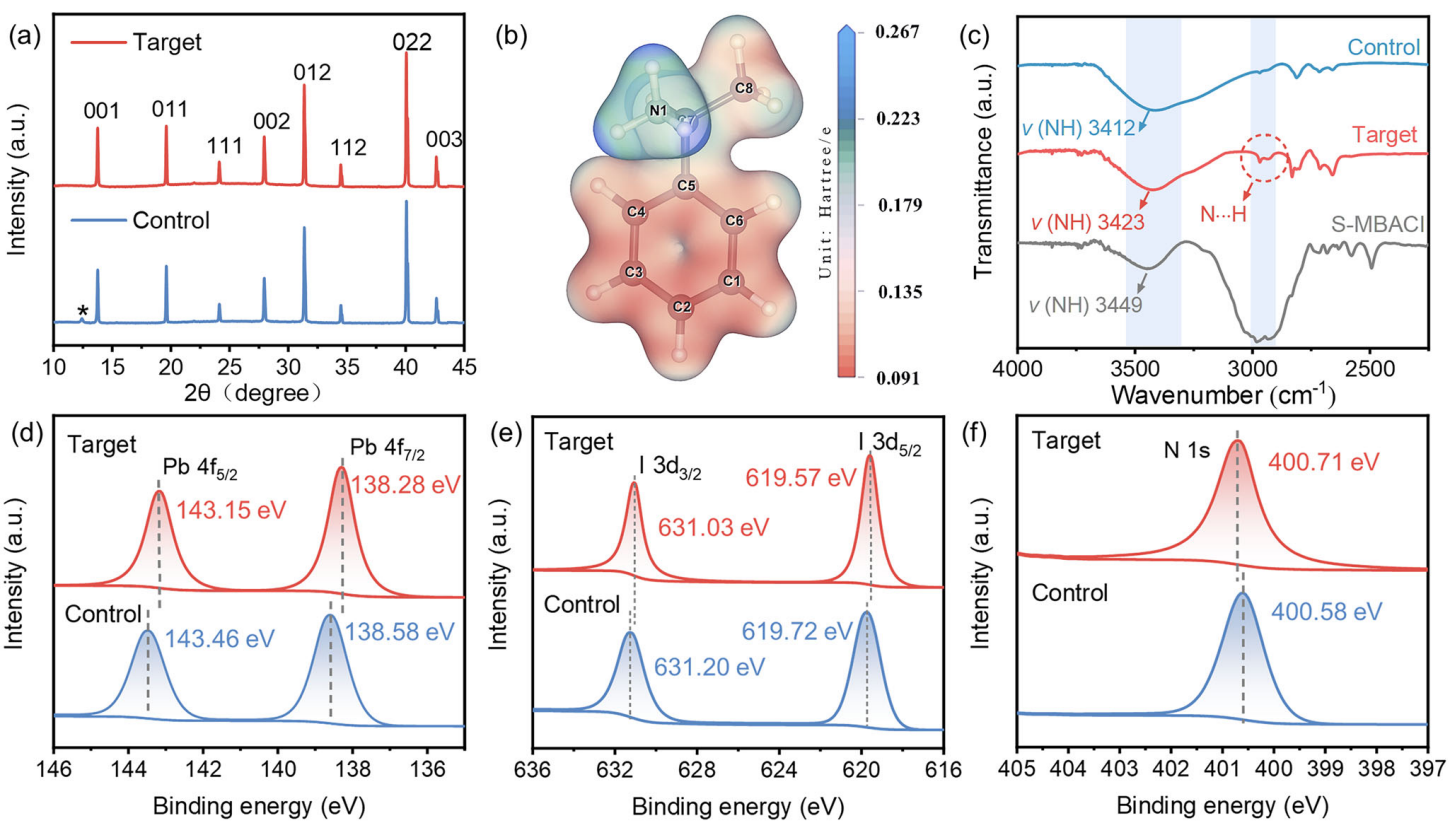
Interaction between the chiral molecule and perovskite surface. a) XRD patterns of perovskite films with and without S‐MBACl modification. b) ESP of S‐MBACl molecule. c) FTIR spectra of perovskite films with and without S‐MBACl modification. XPS profiles of perovskite with and without S‐MBACl modification: d) Pb 4f, e) I 3d, and f) N 1s.

The crystal structures of the perovskite films with and without *S*‐MBACl modification were investigated by performing X‐ray diffraction (XRD). **Figure** **3**a shows that *S*‐MBACl modification significantly enhances the characteristic peak intensity of the perovskite film, indicating higher crystallinity. The intensity of the diffraction peak of PbI_2_ at 12.6° is lower than that of the control sample, indicating that the reaction between the organic amine salt and PbI_2_ is sufficient and no excess PbI_2_ is produced.^[^ ^20^ ^]^ This is beneficial for maintaining device stability. Notably, *S*‐MBACl modification does not result in the emergence of characteristic peaks corresponding to two‐dimensional perovskite phases.^[^ ^21^ ^]^ Figure 3b presents an electrostatic potential (ESP) map of the *S*‐MBACl molecule, which provides an intuitive view of the charge distribution on the functional groups. A higher positive charge density is observed around the ─NH_3_^+^ group, which may enhance the binding strength between the chiral ligands and cation vacancies on the surface of the perovskite. The Fourier transform infrared spectroscopy (FTIR) results depicted in Figure 3c indicate that the characteristic peak of the N–H bond in the modified perovskite sample shifts from 3412 to 3423 cm⁻¹ and moves closer to the N─H peak of *S‐*MBACl at 3449 cm⁻¹. This shift confirms the presence of *S*‐MBACl on the perovskite surface. The *S*‐MBACl peak at ≈3000 cm^−1^ is a frequency‐doubling peak caused by hydrogen bonds between the amino groups. The target sample also exhibits corresponding signal peaks, further substantiating the hydrogen bonding between *S*‐MBACl and the perovskite.

X‐ray photoelectron spectroscopy (XPS) characterization tests were conducted to further confirm the strong interaction between *S*‐MBACl and the perovskite film (Figure S5, Supporting Information). As shown in Figure 3d, the binding‐energy peaks of Pb4f_5/2_ and 4f_7/2_ in the *S*‐MBACl‐modified perovskite film shift toward lower values (decreasing from 143.46 and 138.58 eV to 143.15 and 138.28 eV, respectively), indicating a strong chemical interaction between Pb^2+^ and *S*‐MBACl. This is attributed to the lone‐pair electrons present in the amine group (─NH_3_) of *S*‐MBACl, which can interact with uncoordinated Pb^2+^ to passivate defects and inhibit ion migration. Thus, the electron density around the Pb atoms increases, causing the corresponding Pb 4f signal peaks to shift toward lower *E*_b_ values.^[^ ^22^ ^]^ Figure 3e shows that the binding‐energy peaks of I 3d_3/2_ and I 3d_5/2_ shift toward lower values (from 631.20 and 619.72 eV to 631.03 and 619.57 eV, respectively), confirming the formation of hydrogen bonds (N─H···I) between I in the perovskite and N─H in *S*‐MBACl, which reduces I defects.^[^ ^23^ ^]^ Simultaneously, the N 1s peak shifts toward higher binding energy, indicating a decrease in the electron cloud density in the outer layer of the N atom. This is consistent with the inference that N─H forms hydrogen bonds with iodine.^[^ ^24^ ^]^ Therefore, these results collectively indicate that the incorporation of *S*‐MBACl facilitates strong interactions with the perovskite materials and effectively passivates surface defects.

### 2.4 Effect of Chiral Materials on Energy Properties

**Figure 4:**
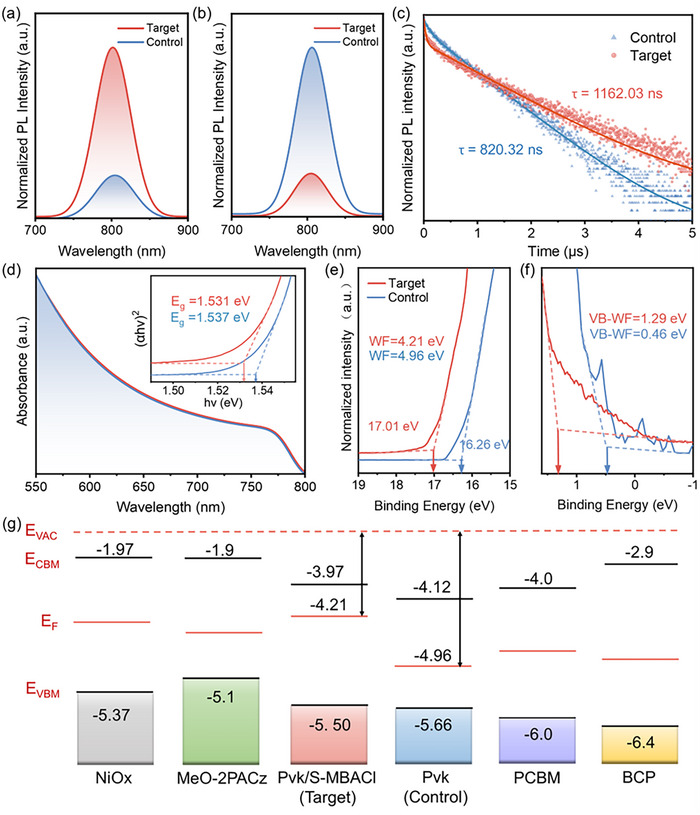
Characterization of optical properties of perovskite films. Steady‐state PL spectra of perovskite films a) without and b) with the addition of a hole‐transport layer. c) TRPL spectra of perovskite films. d) UV–vis spectra of perovskite films. UPS profiles of perovskite films with and without S‐MBACl modification: e) near the secondary electron cutoff region (W_F_: work function) and f) in the valence band (*V*_B_) region. g) Schematic of energy levels. E_F_ and E_VAC_ represent the Fermi and vacuum levels, respectively. E_VBM_ and E_CBM_ denote the energy of the valence band maximum and conduction band minimum, respectively.

The steady‐state optical properties and fluorescence carrier dynamics of perovskite thin films can be further investigated by performing photoluminescence (PL) and time‐resolved PL (TRPL) spectroscopies. The PL test excitation direction is from the front side, which is the halide perovskite side. **Figure** **4**a shows that the PL intensity of the perovskite films modified with *S*‐MBACl is significantly enhanced compared with that of the control films, indicating that defects are reduced and non‐radiative recombination is effectively inhibited. Furthermore, the addition of MeO‐2PACz as a hole‐transport layer significantly decreases the PL intensity, indicating a higher efficiency of hole‐carrier extraction from the *S*‐MBACl‐modified perovskite layer to the SAMs (Figure 4b).^[^ ^25^ ^]^ As shown in Figure 4c, the TRPL measurement results show that the average carrier lifetime of the *S*‐MBACl‐modified perovskite film is *τ*_ave_ = 1162.03 ns, which is higher than that of the control film (*τ*_ave_ = 820.32 ns). This indicates that fewer photon‐generated carriers are trapped and non‐radiative recombination is inhibited.^[^ ^26^ ^]^

Ultraviolet‐visible spectroscopy, as shown in Figure 4d, indicates that the bandgap of the original perovskite, calculated using the Tauc plot method, is ≈1.537 eV, which is consistent with the values reported in previous studies.^[^ ^27^ ^]^ The bandgap of the perovskite thin film modified with *S*‐MBACl decreases slightly from 1.537 to 1.531 eV. Ultraviolet photoelectron spectroscopy (UPS) results reveal a significant improvement in the interface energy‐level alignment. Figure 4e shows that the work function (W_F_) of the control sample is 4.96 eV, which decreases to 4.21 eV after modification with *S*‐MBACl. The conduction band minimum (C_BM_) shifts from −4.12 to −3.97 eV and moves closer to the lowest unoccupied molecular orbital (LUMO) of PCBM (Figure 4f,g).^[^ ^28^ ^]^ Thus, modification with chiral *S*‐MBACl molecules leads to a reduction in the work function of the perovskite surface and a notable increase in the conduction band, thereby establishing a more favorable gradient for energy‐level alignment. This effectively suppresses non‐radiative recombination and enhances electron‐extraction efficiency.

### 2.5 Effect of Chiral Molecules on Device Performance

**Figure 5:**
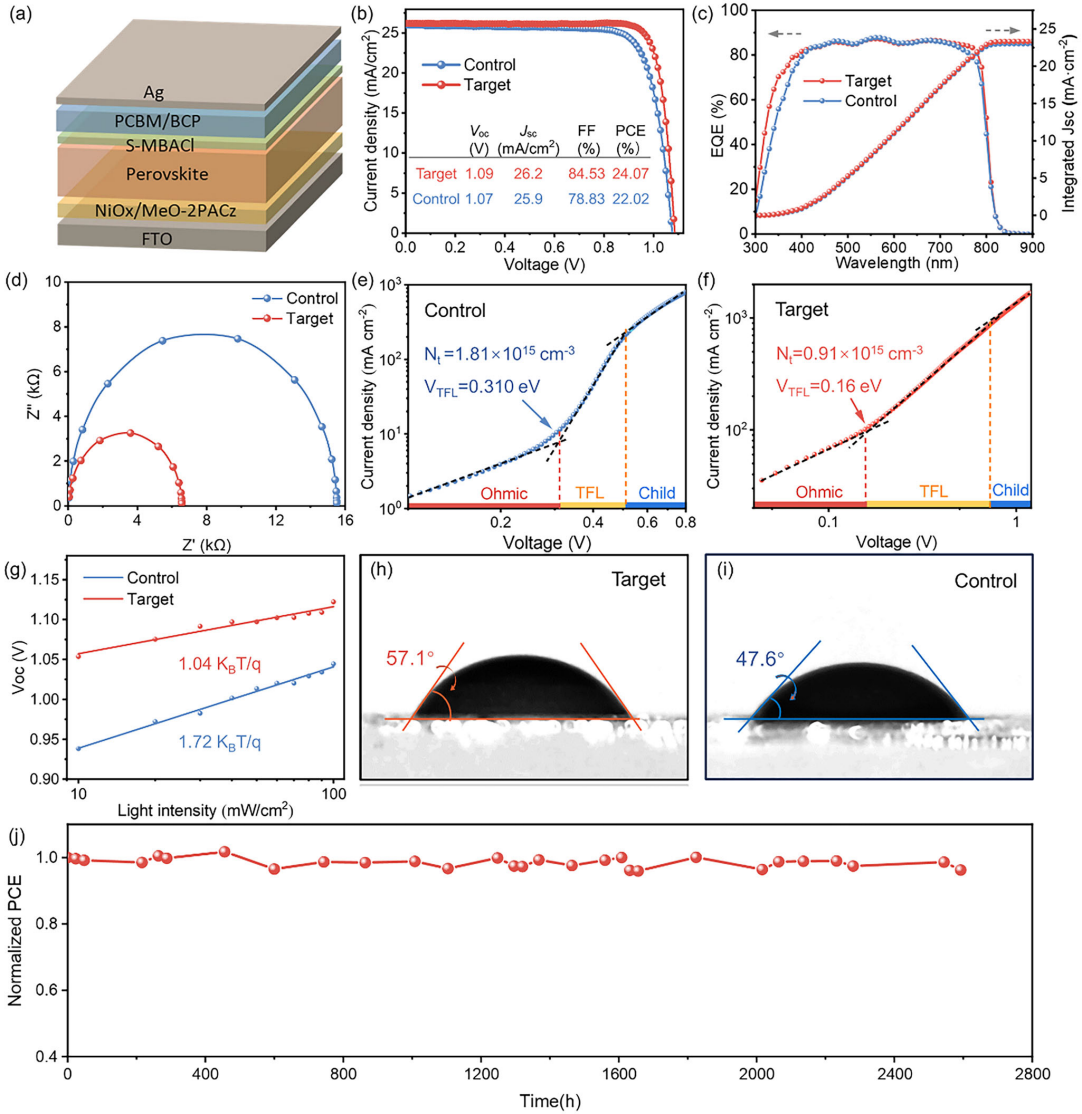
Electrochemical performance and stability of the devices. a) Device structure of the PSC. b) *J*−*V* curves of the devices with and without S‐MBACl modification. c) EQE spectra and d) Nyquist plots of the devices with and without S‐MBACl modification. e) SCLC of the control device. f) SCLC of the S‐MBACl‐modified device. g) Measurement of *V*_oc_ dependence on light intensity. h) Water contact angle of the control sample. i) Water contact angle of the S‐MBACl‐modified perovskite film.

PSCs having the device structure shown in **Figure** **5**a were fabricated to investigate the impact of the chiral ligand molecules on the photoelectric performance of perovskite devices. Figure 5b presents the *J*–*V* curves of the devices before and after modification with *S*‐MBACl.^[^ ^29^ ^]^ After modification with the chiral ligand, the PCE increases from 22.02% to 24.07%. The *J*_SC_, *V*_OC_, and FF also increase primarily because of the reduction in the number of interface defects. We also fabricated the micromodules with an aperture area of 10.62 cm^2^, resulting in a PCE of 19.7% (Figure S6, Supporting Information). The external quantum efficiency (EQE) spectrum is shown in Figure 5c; the integrated current density of the device after *S*‐MBACl modification increases from 22.98% to 23.33%, consistent with the *J–V* curve performance. The electrochemical impedance spectroscopy results in Figure 5d display that the charge‐transfer resistance (*R*_tr_) of the target device is substantially lower than that of the control device, indicating that *S*‐MBACl can promote charge transfer and reduce interfacial charge recombination.^[^ ^30^ ^]^ Using the space‐charge‐limited current (SCLC) method with the device structure of FTO/NiOx/FACsMAPbI_3_/PTAA/Ag, we evaluated the defect density of the films before and after modification (Figure 5e,f).

(1) $$N_{t}=\frac{2\varepsilon \varepsilon_{0}V_{TFL}}{eL^{2}}$$

where *N_t_*  is the density of the defect state, *ϵ *and *ϵ_0_* are the dielectric constant of the material and the vacuum permittivity, respectively, *V*_TFL_  is the trap‐filled limit voltage, *e* is the unit charge, and *L *is the thickness of the perovskite film. The defect density of the target perovskite device is 0.91 × 10^15^ cm^−3^, which is significantly lower than that of the control sample (1.81 × 10^15^ cm^−3^). This indicates that the defect density of the perovskite is significantly reduced and the device quality is improved after modification with *S*‐MBACl.^[^ ^31^ ^]^

As shown in Figure 5g, the device modified with *S*‐MBACl exhibits a lower *V*_OC_ dependence on the light intensity (1.04 k_B_ *T*/q) than the control sample (1.72 k_B_ *T*/q). Here, *k*_B_ is Boltzmann's constant, *T* is the temperature, and *q* is the unit charge. The deviation in the slope of *k*_B_ *T*/q reflects the trap‐assisted recombination in the device.^[^ ^32^ ^]^ These results further confirm that recombination in the perovskite layer of the target sample is largely suppressed. Figure 5h,i shows that the water contact angle of the perovskite modified with *S*‐MBACl (57.1°) is significantly higher than that of the control sample (47.6°), presumably because the ammonium group (NH_3_^+^) of *S*‐MBA^+^ interacts with the surface or grain boundaries of the perovskite and exposes the bulky hydrophobic aromatic rings that can effectively block the penetration of water molecules; thus, the moisture resistance of the perovskite film is enhanced. This is crucial for improving the long‐term stability and performance of PSCs. As shown in Figure 5j, the PCE of unencapsulated *S*‐MBACl‐modified PSCs remains at 89% of the initial PCE after aging at 25 °C for 2400 h in the N_2_ atmosphere.

## 3 Conclusion

This study presents a strategy for passivating defects at perovskite interfaces using chiral molecules. A comparison between the chiral *R*‐, *S*‐, and *rac*‐MBACl molecules reveals that *S*‐MBACl exhibits the strongest chelating ability and defect passivation effect on the perovskite surface. Characterization results indicate that the chiral *S*‐MBACl molecule not only effectively binds to the uncoordinated Pb^2^⁺ on the perovskite surface through its functional groups but also forms strong hydrogen bonding; thus, *S*‐MBACl considerably reduces the defect density in the perovskite film. The addition of *S*‐MBACl significantly prolongs the carrier lifetime and diffusion length, optimizes the energy‐level alignment between the perovskite and charge‐transport layers, and facilitates efficient carrier extraction. In situ KPFM measurements further confirm that *S*‐MBACl promotes electron and hole transport separation, which manifests as a significant increase in the surface potential difference between the dark and illuminated states of the perovskite film. Finally, PSCs based on *S*‐MBACl modification achieve a PCE of 24.06%. After the PSCs are stored under N₂ atmosphere at 25 °C for a certain period, their PCE remains high. Thus, this study not only reveals the important role of chiral *S*‐MBACl molecules in passivating defects on the perovskite surface but also provides a valuable basis for the subsequent exploration of other chiral passivation molecules, thereby opening up new avenues for the performance optimization of PSCs.

## Conflict of Interest

The authors declare no conflict of interest.

## Author Contributions

Z.S. and J.H. contributed equally to this work. J.J.Z. and M.X.W. conceived and supervised the research. Z.X.S. and J.B.H. fabricated the devices and completed the photoelectric performance testing. H.L.D. and Z.Q.C. conducted the GIWAXS test. M.X.L. and Q.R.Z. performed material characterizations. J.J.Z., M.X.W., Z.X.S., and J.B.H. analyzed the test data. Z.X.S. wrote the original draft. J.J.Z. and M.X.W. guided and revised the manuscript. All authors discussed the results and contributed to the writing of the paper.

## Supporting information



Supporting Information

## Data Availability

The data that support the findings of this study are available in the supplementary material of this article.
