# Supplementary material for: Constructing High‐Performance Inverted Perovskite Solar Cells Using Chiral Organic Molecules
Source: Adv Sci (Weinh). 2025 Apr 1;12(22):2417550. doi: 10.1002/advs.202417550 (PMC12165119; doi:10.1002/advs.202417550)
Supplement: Supplementary file 1 — Supporting Information [file ADVS-12-2417550-s001.docx]

Supporting Information

**Constructing High-Performance Inverted** **Perovskite Solar Cells Using Chiral Organic Molecules**

*Zixuan Shang,^1^**^,†^* *Jinbao Han,^1,†^ Hongliang Dong,^2,3^ Mengxi Lv,^1^ Qianru Zhang,^1^ Zhiqiang Chen,^2^ Mingxing Wu,^1, *^Jinjin Zhao^1, *^*

1. Hebei Technology Innovation Center for Energy Conversion Materials and Devices, Hebei Key Laboratory of Inorganic Nanomaterials, Engineering Research Center of Thin Film Solar Cell Materials and Devices, Hebei province, College of Chemistry and Materials Science, Hebei Normal University, Shijiazhuang, Hebei, 050024, China.

2. Center for High Pressure Science and Technology Advanced Research, Pudong, Shanghai 201203, PR China.

3. Shanghai Key Laboratory of Material Frontiers Research in Extreme Environments (MFree), Shanghai Advanced Research in Physical Sciences (SHARPS), Pudong, Shanghai 201203, P.R. China

†These authors contributed equally: Zixuan Shang and Jinbao Han

*Corresponding author. Email: jinjinzhao2012@163.com (J. Zhao)，mingxing.wu@hebtu.edu.cn (M.X. Wu).

**Materials and methods**

**Materials**

Formamidine hydroiodate (FAI, 99.5 %), methyl ammonium iodide (MAI, 95 %), lead iodide (PbI2, 99.9 %), methyl ammonium chloride (MACl, 99.5 %), (6,6) -phenyl carbon 61 methyl butyrate (PCBM), bathocuproine (BCP) and nickel oxide particles (NiOx) were purchased from Preferred Technology Co., LTD. [2(3, 6-dimethoxy-9h-carbazol-9-yl) ethyl] phosphonic acid (MeO-2PACz) was purchased from Japan TCI Company. Solvents including anhydrous ethanol, acetone and isopropyl alcohol (IPA) were purchased from Sinopharm Group Chemical Reagents Co., LTD. (China). Dimethyl formamide (DMF), dimethyl sulfoxide (DMSO) and (R) - (+) - alpha methyl benzyl amine (> 99 %), (S) - (-) - alpha methyl benzyl amine (> 99 %) were purchased from Sigma-Aldrich. Chlorobenzene (CB) was purchased from J&K(China). High purity silver granules were purchased from Beijing Zhongke Yanuo New Material Technology Co., LTD. FTO (8Ω sq^-1^) glass substrate with a thickness of 2.2 mm was purchased from Suzhou Sunyang Technology Co., LTD. (China).

**Preparation of chiral ammonium iodide**

Taking the synthesis of S-MBACl as an example, 6.43 mL (0.05 mol) of (S)-(+)-alpha- methylbenzylamine was added to a wide-mouth bottle, and then 5 mL of anhydrous ethanol was added to dissolve it fully. The wide-mouth bottle was put into an ice bath containing a mixture of ice water and stirred at 0 ℃ for 20 min. Under the condition of avoiding light, 8.0 ml (0.05 mol) of concentrated hydrochloric acid was slowly added and stirred for 2 h. Put the solvent into a vacuum drying oven to evaporate and get the crude product. Add 3 mL of anhydrous ethanol into the solid of the crude product, heat and stir at 80 ℃ until dissolved, and put into the upper layer of the refrigerator (2-8 ℃). The recrystallized product was washed with ether for many times and finally dried to obtain pure S-MBACl. R-MBACl is synthesized in the same way as above. Rac-MBACl is a racemic mixture of equal amounts of R-MBACl and S-MBACl.

**Preparation of perovskite solar cells (PSCs)**

Glass /FTO substrate (8Ω sq^-1^) Ultrasonic cleaning with detergent, deionized water, anhydrous ethanol, acetone and isopropyl alcohol in sequence for 20 minutes. Store in isopropyl alcohol after cleaning. Blow dry with an air dryer and place in a UV ozone cleaner for 25 minutes before use. NiOx nanoparticles were dispersed in deionized water and ultrasonic treated for 5 minutes to obtain NiOx aqueous solution with a concentration of 10 mg ml^-1^. The NiOx was dissolved in water and coated on the FTO substrate for 30 s at 2000 rpm, and annealed at 150 ℃ in ambient air for 15 min. MeO-2PACz was dissolved in ethanol at 0.5 mg ml^-1^ and stirred at room temperature for 3 h. The prepared SAM solution was rotatively coated on the FTO substrate for 30 s at 4000 rpm, and then annealed at 100 ℃ in a nitrogen glove box for 10 min. For the preparation of perovskite precursor solution, it is necessary to add PbI_2_: FAI: MAI: CsI (molar ratio of 1.65:1.27:0.15:0.075), DMF and DMSO (volume ratio of 4:1) according to the chemical formula Cs_0.05_FA_0.85_MA_0.1_PbI_3_, to prepare 1.5 M perovskite precursor solution. 15 mol% MACl was added to control the growth of perovskite, and was heated and stirred in a nitrogen glove box at 60° for 2 h. First, the perovskite precursor solution was spin coated on the hole transport layer at a speed of 1000 rpm /min for 10s, and then spin for 40 s at a high speed of 5000 RPM. 7 s before the end, 150 μl of CB was dropped. The coating was immediately transferred to the hot table and annealed at 100°C for 30 minutes. For devices with surface passivation treatment, dissolve S-MBACl (1 mg ml^-1^) and R-MBACl (1 mg ml^-1^) in IPA solvent separately. A solution of rac-MBACl (1 mg ml^-1^) is obtained by mixing IPA solutions of S- MBACl and R-MBACl in equal parts. The solutions of S-MBACl, R-MBACl and rac-MBACl were then rotated on the prepared perovskite film at 4000 RPM for 30 s and annealed at 100℃ for 5 min. After cooling to room temperature, PCBM (20 mg l^-1^ dissolved in CB) and BCP (2 mg l^-1^ dissolved in IPA) were spin coated on the sample surface at 1500 rpm/min, 30 and 4500 rpm/min, 30 s, respectively. Finally, the 100 nm silver electrode was evaporated at a rate of 1.0 A s^-1^ under high vacuum (< 9 × 10^−4^ units).

**Characterizations**

Time-resolved photoluminescence (TRPL) and steady-state photoluminescence were measured at an excitation wavelength of 375 nm. The steady-state fluorescence spectrum (PL) was measured using the Edinburgh FS5 spectrometer (UK). Time-resolved photoluminescence (TRPL) spectra were measured with the Edinburgh FLS-1000 (UK). The structure analysis and phase characterization of perovskite materials were performed with X-ray diffractometer (XRD) of Rigaku SmartLab 9 kW (Japan) model at a scanning speed of 5°/min and a scanning range of 5°~50°. Fourier transform infrared spectroscopy (FTIR) is determined by Thermo SCIENTIFIC's Fourier Transform Infrared Spectrometer (USA) by scraping the film from the substrate to get a powder sample, and then using KBr particle press to amplify the FTIR spectral signal. The surface and cross section morphologies of perovskite films were obtained by scanning electron microscopy (SEM) (Thermo Scientific Helios 5 CX). Ultraviolet-visible absorbance (UV-vis) was determined using the Agilent Cary 5000 UV-Visible spectrophotometer (USA). XPS measurements were performed on Thermo Scientific ESCALab QXi using 284.8 eV carbon source C1s spectral lines as an energy reference. The ultraviolet photoelectron spectroscopy (UPS) measurements were tested using ThermoFisher Nexsa (UK) with He (21.22 eV) emission lines for excitation. The photocurrent density-voltage (J-V) curve was measured using a Keithley 2400 source meter and simulated sunlight (AM 1.5G,100 mW·cm^-2^), and the illumination intensity of the light source was precisely calibrated using a standard Si solar cell with a KG-5 filter calibrated by NREL, which was tested in a nitrogen glove box. The Kelvin Probe Atomic Force Microscope (KPFM) test used an atomic force microscope built by NT of Russia and a high-precision HA-FM/Pt conducting probe. The bright state was illuminated using a purple LED with a wavelength of 405 nm. The Space Charge Limited Current Test (SCLC) uses the pure electronic structure of FTO/NiOx/PVK/PTAA/Ag to measure the carrier mobility of a material by the relationship between the current density and voltage under an electric field.


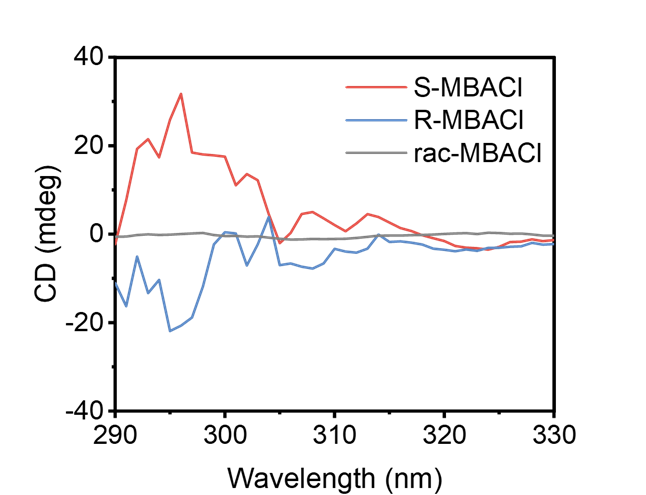


Figure S1. Circular Dichroism (CD) spectra of S/R/rac-MBACl.


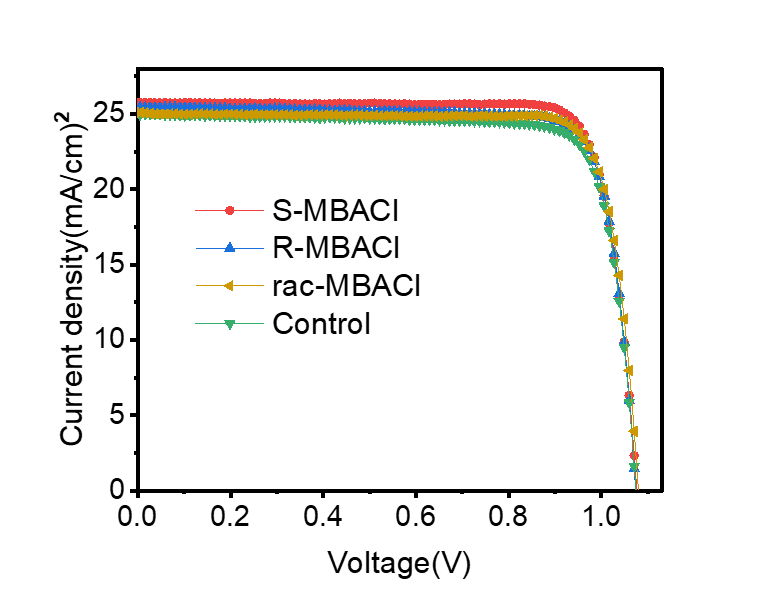


Figure S2. J-V curves of devices before and after modification of S-MBACl, R-MBACl, and rac- MBACl


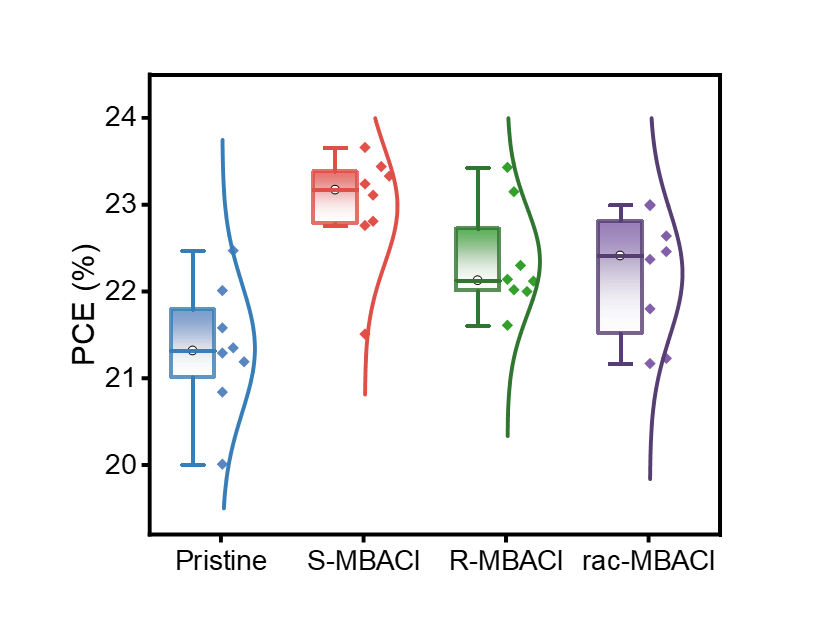


Figure S3. Box plots of the PCE for perovskite solar cells unmodified and modified with S, R, and rac-MBACl, along with their corresponding normal distribution curves.


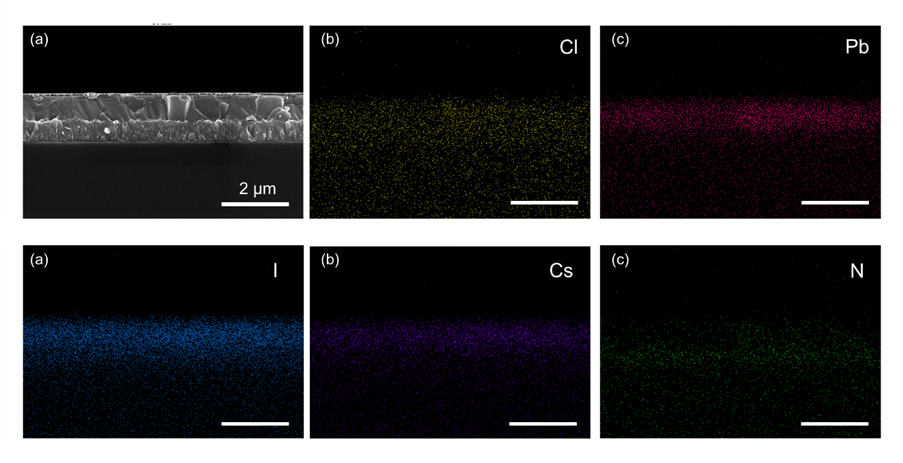


Figure S4. SEM cross section and EDS element Mapping of S-MBACl modified devices


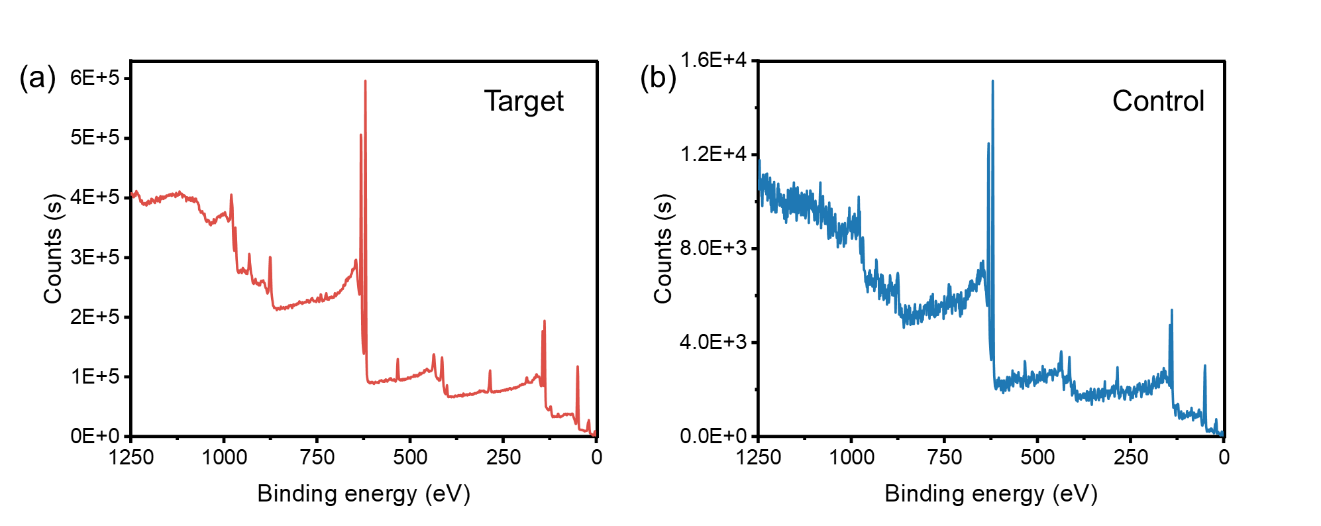


Figure S5. XPS survey spectra of (a) S-MBACl-modified and (b) control perovskite films.


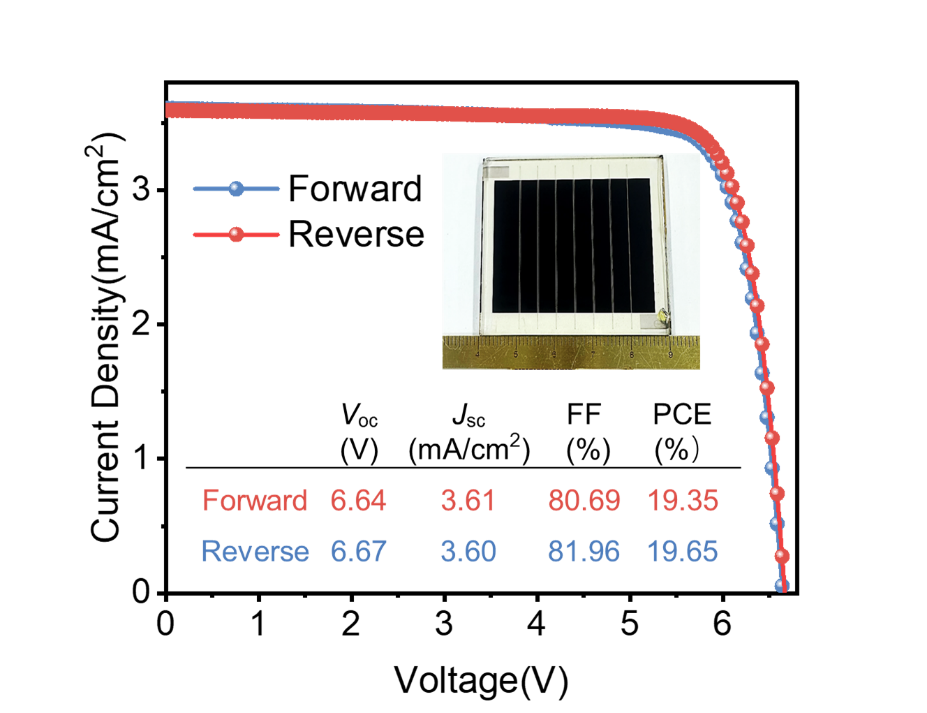


Figure S6. J-V curves of the modulated devices (10.62 cm^2^) after modification of S-MBACl
